# Clarifying the relationship between green investment, technological innovation, financial openness, and renewable energy consumption in MINT

**DOI:** 10.1016/j.heliyon.2023.e21083

**Published:** 2023-10-16

**Authors:** Md Qamruzzaman, Salma Karim

**Affiliations:** School of Business and Economics, United International University, Dhaka, 1212, Bangladesh

**Keywords:** Renewable energy consumption, Green investment, Technological innovation, Financial openness, Cross-sectional autoregressive distributed lagged, Nonlinear autoregressive distributed lagged

## Abstract

The importance of Renewable energy has been well documented in the literature, especially in the nexus of renewable energy-led environmental sustainability. The purpose of the study is to gauge the effects of green investment (GI), technological innovation (TI), and financial Openness (FO) on Renewable Energy Consumption (REC) in MINT for the period 1996–2019. Several econometric tools have been considered in documenting the target nexus, including the panel unit root test following CADF and CIPS, Error correction Cointegration test, CS-ARDL, nonlinear ARDL, and directional causality test by employing the D-H Causality test. The panel unit root test revealed that all the variables become stationary after the first difference. The long-run association in the target model is unveiled with the panel cointegration test. A positive and statistically significant connection regarding FO, TI, and GI coefficients on REC has been exposed. It suggests that the progress in RE development and inclusion in economic activities could be amplified through FO, TI, and GI. Inferring the results of asymmetric valuation, the test statistics of a standard Wald test document asymmetric association in the long run and short run. Furthermore, the coefficients of positive and negative innovation in explanatory variables, i.e., TI, GI, & FO, divulge a positive statistically significant tie to REC, which is valid in long-run and short-run assessment.

## Highlights

• The nexus between GI, TI, FO and REC has assessed.
• The study applied CS-ARDl and Nonlinear ARDL.
• Study revealed positive tie between explanatory and REC.
• DH causality test documented feedback hypothesis.

## 1 Background of the study

Because of rising worries about climate change and the stability of their energy supplies, several countries are looking at methods to cut greenhouse gas emissions while still ensuring the security of their energy supply. All are within reach with the support of renewable energy sources, environmentally sound development, decreased dependence on imported resources, and the capacity to meet the energy demands of a thriving economy. Many countries are starting to include the development of renewable energy sources in their overall energy policies. Since the passage of the Kyoto Protocol in 1997, several countries have gradually created a series of energy policies promoting the expansion of renewable energy sources. Renewable energy consumption increased by 1.89% from 1990 to 2009. According to International Energy Agency predictions, renewable energy consumption will grow by 3.13% annually between 2009 and 2035. (IEA). According to the International Energy Agency (IEA), renewable energy consumption in MINT nations (Mexico, Indonesia, Nigeria, and Turkey) has increased recently. However, it remains relatively low compared to other regions. In Mexico, renewable energy consumption has grown significantly in the past decade, from less than 5 % in 2010 to 9 % in 2019 [1].

Most of this growth has been in wind and solar power, which have benefited from government policies promoting renewable energy development. Renewable energy consumption in Indonesia remains low, at less than 5 % in 2019. The country has significant potential for renewable energy development, with abundant solar, wind, and geothermal resources. However, the high cost of renewable energy infrastructure and limited government support have hindered its growth. Renewable energy consumption in Nigeria is also relatively low, at around 2 % in 2019. The country has significant potential for solar and wind power. However, it faces challenges in developing renewable energy infrastructure due to limited funding, regulatory barriers, and public awareness. REC in Turkey has grown significantly in recent years, from less than 10 % in 2010 to 22 % in 2019. The country has prioritized renewable energy development, particularly wind and solar power. It has implemented policies to support the growth of the sector. While renewable energy consumption in MINT nations has grown in recent years, there is still significant room for growth. These countries have abundant renewable energy resources but face challenges in developing the necessary infrastructure and policies to support their growth. By implementing the policy suggestions outlined in our previous response, these countries can accelerate the adoption of renewable energy and reduce their carbon footprint [[2], [3], [4]].

Consequently, renewable energy's share of overall energy consumption will climb from 13 % in 2009 to 18 % in 2035. Its share of global power output will rise from 18 % in 2009 to 30 % in 2035. Energy consumption in many countries, particularly emerging economies, has expanded faster than domestic energy output [5]. Because of mounting worries about global warming caused by our continued dependence on nonrenewable energy sources and the depletion of natural resources, numerous countries are shifting their focus to renewable energy sources [6]. Pollution in the environment is increasing due to increased industrialization and human work. The drive for renewable energy sources, energy uses, and smart grid technology is centred on sustainable development [7]. Environmental economics is interested in the debate over the advantages and disadvantages of adopting renewable energy over fossil fuels. Because of the dynamics of renewable energy, many new ideas, such as trade liberalization, economic development, and technological progress, have evolved in recent years [[8], [9], [10], [11]].

Increasing energy use significantly affects environmental conditions and carbon dioxide emissions [12]. Compared to fossil fuels, which are widely acknowledged as the primary contributor to climate change and CO2 emissions [13,14], renewable energy sources (including solar, tidal, geothermal, wind power, biomass, and hydro) are known to yield lower levels of greenhouse gas emissions, and this is why switching to renewable energy is so essential for the environment. Developed countries that want to reduce their greenhouse gas emissions increasingly turn to renewable energy sources after the globally recognized Kyoto Protocol in 2005 and the United Nations Conference on Climate Change (COP-21). Conventional energy sources and renewables do not contribute to environmental degradation or global warming. Several studies have included renewable energy's incorporation as a significant variable in CO2 emissions regressions due to the significance of doing so in reducing atmospheric concentrations of carbon dioxide [15].

The present study has considered green investment, technological innovation, and financial openness in the Equation of renewable energy consumption (REC, hereafter) in MINT for 1990–2019. The four countries that make up the MINT group—Mexico, Indonesia, Nigeria, and Turkey—stand out among the emerging market nations in that they are responsible for an increasing portion of the global carbon dioxide emissions problem. The Intergovernmental Panel on Climate Change (IPCC) concluded that developing market economies prioritized economic growth more than they did the environmental sustainability of their economies, demonstrated by the fact that these economies grew while emitting over 76.6% of the world's greenhouse gas (GHG) emissions, most of which were CO2. Even though developed countries are primarily responsible for global warming, developing economies' contribution to total carbon dioxide emissions has grown over the last several years. According to existing literature**, Public** investment in developing renewable energy sources such as wind, solar, geothermal, ocean, biomass, and waste energy might significantly aid in attaining environmental objectives. Using a more significant share of renewable energy might help achieve several public policy goals, including energy security in fluctuating fossil fuel markets [[16], [17], [18], [19]]. Moreover, the influence of financial openness on the proliferation of investments in renewable energy has not gotten much attention from researchers. The dearth of academic studies makes it difficult to comprehend how the two factors are related. The choices for the dependent variables in the few assessments that are currently available have been the consumption and the production of renewable energy, renewable energy technologies, and the installed capacity of renewable energy; on the other hand, financial development, financial flows, and foreign direct investment have been considered FDI [20].

The study possesses several distinctive characteristics that differentiate it from other studies in the field. These attributes contribute to its uniqueness and potential significance. These are the key elements of the study as follows. First, This study adopts a comprehensive perspective by examining various interrelated factors, including green investment, technological innovation, financial openness, renewable energy consumption, remittances, and urbanization. Through an analysis of the relationships between these variables, the study thoroughly explains the intricate dynamics present in the MINT countries. The comprehensive perspective presented in this study sets it apart from others that solely focus on individual factors in isolation. Second, the study presents a comprehensive framework for analyzing the correlation between green investment, technological innovation, financial openness, and renewable energy consumption. Through the process of integrating these dimensions, the study aims to elucidate the intricate interplay and interdependencies that exist among these factors. The utilization of this framework enhances the overall resilience of the study. It ensures a thorough analysis of the topic at hand. Third, the research specifically focuses on the MINT nations: Mexico, Indonesia, Nigeria, and Turkey. These nations represent diverse emerging economies, each with distinct characteristics, challenges, and opportunities. By confining its scope to these particular nations, the study offers valuable, particularly relevant insights. The regional concentration of this study sets it apart from more generalized analyses encompassing a broader range of countries or regions. Fourth, The research delves into factors beyond the conventional variables typically analyzed in studies about green investment and renewable energy consumption. This study examines the influence of remittances and urbanization on the relationship between these variables. The study sheds light on previously unexplored aspects by incorporating these innovative dimensions. It enhances our understanding of the topic at a more profound level. Combining these characteristics collectively enhances the study's potential to generate innovative insights, propel the field forward, and provide valuable guidance for future research endeavours and policy-making [21].

The contribution of the study is as follows. *First*, the growth of renewable energy sources demands substantial capital investment from home and abroad, governmental policy assistance, industrial structure, and energy accessibility. According to the existing literature, public and private investments significantly affect energy progress and transition. However, the promotion and implementation of green investment in renewable energy sources have yet to be investigated extensively. Moreover, according to the literature, this is the first-ever empirical study for MINT nations where the effects of Green investment have been assessed for renewable energy consumption. *Second*, the effects of explanatory variables green investment, technological innovation, and financial openness on renewable energy have been investigated by employing the decomposition of asymmetric shocks over the conventional investigation. The critical limitation of the conventional assessment is that it only offers an avenue for one-directional assessment. At the same time, the asymmetric shocks inclusion in empirical investigation exposed the elasticities of explanatory variables with positive and negative changes. The outcome of positive and negative changes gently supports policy formulations over conventional analysis. *Third*, as a determinant of renewable energy, the impact of green investment, technological innovation, and financial openness have been investigated scattered. The study investigated the empirical nexus between explanatory variables, i.e., GI, TI, and FO, and renewable energy consumption in a single equation with the framework of symmetric assessment by employing CS-ARDL and Nonlinear ARDL.

The motivation of the study is to gauge the potential effects of GI, TI, and FO on REC in the MINT economy for the period 1995–2019. Both symmetric and asymmetric frameworks have implemented the association between explained and explanatory variables and their elasticities of **renewable energy consumption**. The results of the slope of homogeneity and cross-sectional dependency test revealed that research units possessed heterogeneity properties and were cross-sectionally dependent. The results of the panel unit roots test, CADF, and CIPS disclosed that the variables become stationary after the first difference, suggesting the variables' order of integration after the first difference, i.e., I(1). Second, the output from the pane cointegration test dealing with determining the long-run association between explained and explanatory variables has revealed statistical significance at a 1 % level, suggesting a long-run relation in the empirical Equation. Third, according to the coefficients derived from CS-ARDL estimation, GI, TI, and FO on REC have exposed positive and statistically significant at a 1 % level in the long-run and short-run assessment. This advocates the beneficial effects of GI, TI and FO in the clean energy transition from conventional to renewable sources. The nonlinear assessment revealed an asymmetric association both in the long-run and short-run horizon. Regarding the asymmetric coefficients of GI, TI, and FO on REC, the study unveiled a positive and statistically significant linkage, implying that any fluctuation in explanatory variables would result in REC movements. Fourth, the directional casualty results in the feedback hypothesis hold between green investment and renewable energy (GI←→RE); globalization and renewable energy [GLO←→RE]; financial development and renewable energy [FD←→RE]. Furthermore, the unidirectional causality documented between renewable energy consumption and financial openness [REC→FO], technological innovation, and renewable energy consumption [TI→REC].

The remaining structure of the paper is as follows. Focusing on the target nexus, the pertinent literature survey is displayed in Section II. The data, variables definition, and estimating strategies are exhibited in Section III. Empirical model output and its interpretation are available in Section IV. Section V discusses the findings, conclusion, and policy implications in Section VI.

### 1.1 State-of-the-art

#### 1.1.1 Green investment and renewable energy

Investing money in renewable energy sources is beneficial for the environment in the long term, even while the pandemic is still going on [22,23]. Providing environmentally conscious and resource-conserving enterprises with loans at a fixed interest rate is called “green financing.” Companies that provide loans to budding entrepreneurs so they may start firms that use environmentally friendly methods seem to be doing well worldwide. Businesses are providing more and more support for environmentally conscious economic activities [24]. The industrialized world has the necessary infrastructure to support the green economy [25]. The role of green investment in carbon emission has been investigated in the literature. For example, Li, Li [26] evaluated the role of green finance promotion in environmental development by reducing carbon emissions in 30 provinces from 1995 to 2017. The coefficient of green investment through CS-augmented ARDL revealed a negative and statistically significant linkage, suggesting the reduction of carbon emission with the flourishment of green investment both in the long-run and short-run horizon. On the other hand, financial development, extraction of natural resources, and energy investment accelerated carbon emissions.

Considering the 141 publicly listed Chinese renewable energy enterprise-level data group, He, Liu [27] assess the impact of green financial development on the efficiency of investment in the renewable energy sector by employing the Richardson model. The study discovered that China's green financial development negatively impacts bank loan issuance and slows the efficiency gains in renewable energy investment to 0.0017%. The study claims that legislation and oversight from the state are essential to establishing and maintaining a green financial system. Financial institutions that actively produce innovative green financial solutions may help renewable energy enterprises by assisting with internal management and constructing new financing channels. In the case of OECD, Sun, Guan [28] investigated the impact of green investment, renewable energy consumption, and financial decentralization on environmental quality from 1990 to 2019. Cutting-edge panel data estimators validate cross-sectional dependence, unit roots, and cointegration among model variables. In addition to using linear heterogeneous estimators, this study incorporates the asymmetric behaviour of regressors using moment's quantile regression (MMQR) techniques. According to preliminary estimations, the key variables are cointegrated, non-stationary, and interdependent across time. The results imply that fiscal decentralization, green investment, and the usage of renewable energy all have a significant role in lowering ecological footprints, even if the proportionate importance of these factors varies significantly across the lower, middle, and higher quantiles. These impacts are more notable in the middle and upper quantiles. The stark negative association between fiscal decentralization and green investment and the sample nations' significantly reduced ecological footprints. These findings imply that fiscal decentralization may promote environmental sustainability by encouraging green investment and switching to renewable energy sources [29].

#### 1.1.2 Technological innovation and renewable energy

It is widely acknowledged that technological innovation may help reduce negative environmental impacts. Jan, Xin-gang [30] examined the connection between technological advancement and CO2 for nine oil-exporting nations using yearly data from 1990 to 2018. Using cutting-edge econometric methods, the authors probed the nature of this connection. The findings proved that cutting-edge technology improves ecosystem health. Yasmeen, Tan [31] looked at the link between PPIE and CO2 emissions in China, considering the significant role that technical progress plays in this emissions category. The findings show that PPIE reduces productivity, increases waste, and lowers the quality of life, reducing environmental sustainability. The opposite was shown to be accurate; they found that progress in technology increased CO2 emissions. Evidence for a U-shaped connection between natural growth and CO2 pollution supports the EKC hypothesis.

Khan, Su [32] assesses the role of technological innovation in fostering renewable energy consumption in Germany. The study disclosed a positive statistically significant tie between TI and REC; the directional causality revealed bidirectional causality holds between them. On a policy note, the study advocated that the progress of RE development can be intensified through investing in TI. For Brazile, do Valle Costa, La Rovere [33] reveal policy formulation and implementation targeting the acceleration of TI prompts RE inclusion and application in the industrial output? Additionally, they found that exports degrade environmental quality, whereas FDI imports improve it. Using wavelet coherence and ARDL techniques, Adebayo, Oladipupo [34] studied the connections between CO2 emissions, GDP growth, and financial development in Mexico. Their research established a connection between rising GDP and rising carbon dioxide levels. Further, growing economies reduce environmental standards.

The second purview explains the impact of renewable energy on technological innovation. Renewable energy has substantially impacted technological innovation in developing new renewable energy technologies and the economy. Here are a few examples of how renewable energy has influenced technological advancement: Significant investments have been made in research and development (R&D) for new renewable energy technologies due to the growing demand for renewable energy [35]. This has resulted in the development of innovations and advancements in the field of renewable energy, such as enhancements in solar panel efficacy, wind turbine design, and energy storage technology [36,37]. The expansion of the renewable energy industry has created new opportunities for businesses and enterprises, thereby fostering economic expansion and the creation of new jobs, which resulted in new industries, such as the fabrication of solar panels and wind turbines, which require specialized labour and advanced technological expertise. Spillovers of Technology [[38], [39], [40], [41], [42]] Developing renewable energy technologies has also resulted in technological spillovers in other economic sectors. For instance, technological advancements in energy storage have led to the development of novel battery technologies for use in electric vehicles and consumer electronics. Collaboration and Partnerships: The development of renewable energy has encouraged business, government, and research institution collaboration and partnership, has resulted in the sharing of knowledge and expertise, facilitating the creation of innovations and advancements. Renewable energy has promoted sustainable development by reducing greenhouse gas emissions and reliance on fossil fuels. This has resulted in a greater emphasis on sustainability and environmental protection, spurring the development of new technologies and innovations supporting these objectives [43,44]. Renewable energy has significantly impacted technological innovation, spurring new developments and advancements in energy technologies and beyond. As the significance of renewable energy continues to rise, it will likely continue to play an important role in propelling technological innovation in the future [45].

As far as environmental sustainability is concerned, many studies have documented the beneficial role of renewable energy and technological innovation in managing the level of carbon emission in the ecosystem [[46], [47], [48], [49], [50]]. For instance, Dong, Sun [51] examine the nexus between renewable energy, urbanization, and natural gas consumption in China by applying the ARDL approach. The study postulated that renewable energy and natural gas consumption produce a pleasant ambience for environmental quality development by reducing carbon concentration. In MINT countries, Akram, Umar [52] assess the influence of energy efficiency and renewable energy consumption on environmental sustainability and the reduction of CO2 emission for the period 1990–2014 b y employing the nonlinear framework. Inferring the asymmetric assessment, the empirical association has long-run asymmetric cointegration. Referring to both long-run and short-run.

#### 1.1.3 Financial openness and renewable energy consumption

The degree to which a country is open to international financial flows, such as foreign investment and commerce, is referred to as its financial openness. Renewable energy consumption refers to the utilization of replenish able energy sources, such as solar, wind, and hydro power. The relationship between financial openness and renewable energy consumption is complex and varies by country. However, financial openness can positively and negatively affect renewable energy consumption. Positively, financial openness can facilitate the passage of capital into renewable energy initiatives, thereby facilitating their development and deployment. This can be especially significant for developing nations, which frequently lack the financial means to invest in renewable energy infrastructure. On the downside, financial openness can encourage nations to prioritize short-term economic development over long-term environmental objectives. For instance, countries may be more inclined to invest in fossil fuel infrastructure if they believe it will generate a higher rate of return than renewable energy projects. Countries may need to implement policies prioritizing sustainability and environmental protection to maximize the benefits of financial openness while promoting renewable energy consumption. This could include incentives for investment in renewable energy initiatives and regulations that encourage the use of sustainable energy sources while discouraging investment in fossil fuels. In addition, international cooperation and coordination may be required to direct financial flows toward sustainable development objectives.

Many studies have divulged the positive linkage between financial openness and economic growth, suggesting FO accelerated economic progress [2,31,53,54]. In the case of SSA, Gabriel and David [55] investigated the impact of trade openness and financial openness on economic growth in SSA by implementing GMM and thesystem–GMM approach. Study findings established that the magnitude of financial openness on economic growth varies with the economy's economic structure. This implies that TO is positive and significant for lower-income economies with EG, but FO was found statistically insignificant. At the same time, middle and higher-income countries revealed positive and statistically significant. This study advocated for accelerated economic growth by thriving financial openness. As a lower-income economy, SSA has to offer appropriate benefits to entice the inflows of FDI. The research of Murshed, Elheddad [56] makes a novel attempt to assess how FDI inflows affected Bangladesh between 1972 and 2015 regarding increasing the usage of renewable energy sources and achieving a green environment. It has been discovered that FDI inflows increase the percentage of renewable electricity production in the nation's overall electricity output levels. Consequently, it can be concluded that FDI encourages the creation of renewable electricity in Bangladesh but turns the country into a home for pollution.

The research by Azam and Haseeb [57] primarily focuses on the BRICS countries' foreign direct investment (FDI) inflows with a particular focus on the impact of both renewable and nonrenewable energy from 1990 to 2018 in BRICS countries. According to the long-term findings, FDI inflows are favourably impacted by renewable and nonrenewable energy use, where renewable energy has a more significant impact on FDI than nonrenewable energy. The study by Adebayo, Coelho [58] investigates the links between the use of renewable energy and FDI and some other core variables for a panel of 22 Central and South American nations from 1995 to 2010. Long-run estimates for the entire panel and the three income panel groups considered (Lower Middle, Upper Middle, and High) show that mainly renewable energy and FDI help reduce emissions. Therefore, for this region to tackle climate change, increasing foreign direct investment, promoting the use of renewable energy, and developing tourism, especially green tourism, are desirable strategies. The study by Khan, Chenggang [59]addresses the evaluation of the effect of FDI with some crucial variables prominently on renewable energy utilizing 69 countries of BRI from 2000 to 2014. The output of the study conveys that there is a negative affinity between FDI and renewable energy.

Further, the Granger non-causality test shows a two-way causal linkage between these two variables. Hence, funding in R&D is required for those nations for firm policy implementations. The paper of Muhammad, Khan [60] perused the effects of FDI along with several diverse variables, importantly renewable energy consumption and how they are related and exerting an impact on the environment for the BRICS countries from 1991 to 2018. The paper found that FDI is responsible for environmental degradation, whereas renewable energy positively influences environmental quality. The journal by Doytch and Narayan [61] was assessed to introduce the amount of commerce, renewable energy consumption, and FDI from 1995 to 2017 to promote a sustainable environment in China. Results say that FDI, renewable energy consumption, and technological innovation enhance carbon emissions. The paper of Qamruzzaman and Jianguo [62] et al. (2020) attempted to fill the gap by solving the symmetricity of the variables, including FDI and renewable energy, along with other factors using panel nonlinear ARDL model in three subsamples, i.e., lower, middle and upper middle-income countries for the data using 1990 to 2017. The study unveiled that all three countries have asymmetric relationships and directional causality in the long run.

In contrast, only lower-income countries have symmetric relationships in the short term. The research of Koengkan, Fuinhas [63] investigated the financial openness of renewable energy investments employing ten Latin American countries for the period 1980 to 2014. The research witnesses the long-term positive and bidirectional association of these two variables. The journal of Khandker, Amin [64] discussed the catalytical role of FDI with renewable energy in developing countries to stimulate productivity in Bangladesh from 1980 to 2015. As with the results, for emerging countries, increasing the use of renewable energy shrinks the growth of trade openness or FDI. Hence, the journal also discussed effective policies for the authorities to increase the use of renewable energy while encouraging FDI parallelly. The article of Rasoulinezhad and Saboori [65] is about the long-term and causal association of renewable energy, FDI, and some other variables using samples from the Commonwealth of Independent States (CIS) from 1992 to 2015. Engaging Johansen's cointegration and Granger causality test, there is a positive and long-run co-integrated, bidirectional causality in these variables, so attracting more FDI is suggested. The article by Doytch and Narayan [61] investigated the relationship between FDI (splitting into different components) by decomposing energy into renewable and nonrenewable energy usage. 74 nations between 1985 and 2012. The findings appear to propose a reduction in energy consumption for nonrenewable energy sources and an increase in energy consumption for renewable energy sources.

## 2 Model specification, theoretical underpinnings, and estimation strategies

### 2.1 Model specification

The empirical nexus between renewable energy consumption (REC), technological innovation (TI), green investment (GI), and financial openness (FO) in the case of MINT countries for the period can be defined in the following manner.

(1) $${EC}_{REC,}^{i,t}\int {GI}^{it},{TI}^{it},{\begin{array}{c} FO \end{array}}^{it}$$

Moreover, the study has considered a list of control variables following the existing literature [43,[66], [67], [68], [69], [70], [71]], focusing on the determinants of renewable energy. The study considered urbanization, globalization, and financial development, and the above Equation (1) has been extended in the following manner.

(2) $${EC}_{REC,}^{i,t}\int {GI}^{it},{TI}^{it},{\begin{array}{c} FO \end{array}}^{it},{\begin{array}{c} UR \end{array}}^{it},{\begin{array}{c} GLO \end{array}}^{it},{\begin{array}{c} FD \end{array}}^{it}$$

The above Equation (2) can be reported in the predictive framework for further assessment in the following manner.

(3) $${REC}_{it}=\alpha_{0}+\beta_{1t}{GI}_{it}+\beta_{2t}{TI}_{it}+\beta_{3t}{FO}_{it}+\beta_{4t}{UT}_{it}+\beta_{5t}{FD}_{it}+\beta_{6t}{GLO}_{it}+\varepsilon_{t}$$

In equation (3), REC, GI, TI, FO, RU, FD, and GLO stand for renewable energy consumption, green investment, technological innovation, financial openness, urbanization, financial development, and globalization, respectively. The coefficients of explanatory variables and control variables can be obtained from $\beta_{1t}\ldots \ldots .\beta_{6t}$. Table 1 displayed the proxies of research variables and data sources

**Table 1:** Definition and data sources for the research variables.

Variables	Definition	Reference	Sources
Renewable energy consumption	The share of renewable energy in total energy consumption	[43,68,69,72,73]	WDI
Green investment	The study used public investment in renewable energy as a proxy for green investment (GIN)	https://doi.org/10.3390/su132212873 [72]	
Financial openness	the Chinn-Ito index as a proxy for financial openness (FIOPEN		
	*fdigdpit* is the financial openness	[55]	

### 2.2 Estimating strategies

#### 2.2.1 Cross-sectional dependency taste

The Lagrange multiplier (CD_lm_) is the scaled version of the LM test following Pesaran [74], the CD test following Pesaran [75], and Pesaran, Ullah [76] proposed the bais-adjusted LM test, which is preferred in a situation when the cross-section (N) is smaller than time (T). Based on the following Equations (4) and (5), we can construct LM test statistics:

(4) $$y_{it}=\alpha_{i}+\beta_{i}x_{it}+u_{it}\;i=1\ldots \ldots N,t=1\ldots \ldots T$$

(5) $$LM=T\sum_{i=1}^{N-1}\sum_{j=i+1}^{N}{\hat{\rho }}_{IJ\underset{d}{\to }X^{2}N\left(N+1\right)2}$$

The Lagrange multiplier (CD_lm_) is the scaled version of the LM test in Eq (6):

(6) $${CD}_{lm}=\sqrt{\frac{N}{N\left(N-1\right)}}\sum_{I=1}^{N-1}\sum_{J=i+1}^{N}\left(T{\hat{\rho }}_{ij}-1\right)$$

in the case of larger N relative to T, CD*lm* estimation is subject to size dissertation. Therefore, Pesaran [75] proposed the following CD test see Eqs (7), (8), which is suitable in a situation when N is more significant than T:

(7) $${CD}_{lm}=\sqrt{\frac{2T}{N\left(N-1\right)}}\sum_{I=1}^{N-1}\sum_{J=i+1}^{N}\left({\hat{\rho }}_{ij}\right)$$

(8) $${CD}_{lm}=\sqrt{\frac{2}{N\left(N-1\right)}}\sum_{I=1}^{N-1}\sum_{J=i+1}^{N}\left(\frac{\left(T-K\right){\hat{\rho }}_{ij}^{2}-u_{Tij}}{\upsilon_{Tij}^{2}}\right)\vec{d}\left(N,0\right)$$

Where k refers to the number of regresses, $u_{Tij}$ and $\upsilon_{Tij}^{2}$ specifies the mean and variance of $\left(T-K\right){\hat{\rho }}_{ij}^{2}$, respectively.

#### 2.2.2 Panel unit root test: CADF and CIPS

The CADF and CIPS panel unit root tests are two of the most commonly used tests for assessing stationarity in a time series. Both tests are based on the concept of a unit root, a characteristic of a time series that can be used to determine whether it is stationary. The CADF test is based on the idea that a time series with a unit root is non-stationary. In contrast, a time series without a unit root is stationary. The test statistic for the CADF test is calculated as the sum of the absolute values of the autocorrelations at lag 1, 2, 3, and 4. If the test statistic is greater than 2.58, then the null hypothesis of stationarity cannot be rejected at the 5 % level. The CIPS test is based on the idea that a time series with a unit root is non-stationary. In contrast, a time series without a unit root is stationary. The test statistic for the CIPS test is calculated as the sum of the squared autocorrelations at lag 1, 2, 3, and 4. If the test statistic is greater than 16.919, then the null hypothesis of stationarity cannot be rejected at the 5 % level.

The framework for unit root test with CADE following Pesaran [77] are as follows:

(9) $${\Delta Y}_{it}=\mu_{i}+\theta_{i}y_{i,t-1}+\gamma_{i}{\bar{y}}_{t-1}+\vartheta_{i}{\bar{y}}_{t}+\tau_{it}$$

Putting long-term in Equation (9) results in the following Equation (10):

(10) $${\Delta Y}_{it}=\mu_{i}+\theta_{i}y_{i,t-1}+\gamma_{i}{\bar{y}}_{t-1}+\sum_{k=1}^{p}\gamma_{ik}{\Delta y}_{i,k-1}+\sum_{k=0}^{p}\gamma_{ik}{\bar{\Delta y}}_{i,k-0}+\tau_{it}$$

Where $Y_{it}-1$ and ${\bar{y}}_{t-1}$ stands lagged level average and first difference operator for each cross-section, the CIPS unit root test displays in equation (11).

(11) $$CIPS=N^{-1}\sum_{i-1}^{N}\partial_{i}\left(N,T\right)$$

Where the parameter $\partial_{i}\left(N,T\right)$ explain the test statistics of CADF, which can be replaced in the following manner:

(12) $$CIPS=N^{-1}\sum_{i-1}^{N}CADF$$

#### 2.2.3 Westerlund cointegration test

The error correction techniques for long-run cointegration assessment are as follows:

(13) $${\Delta Z}_{it}=\partial_{i}^{\prime }d_{i}+\emptyset_{i}\left(Z_{i,t-1}-\delta_{i}^{\prime }W_{i,t-1}\right)+\sum_{r=1}^{p}\emptyset_{i,r}\Delta Z_{i,t-r}+\sum_{r=0}^{p}\gamma_{i,j}{\Delta W}_{i,t-r}+\epsilon_{i,t}$$

The results of group test statistics can be derived with equations (14) and (15).

(14) $$G_{T}=\frac{1}{N}\sum_{i-1}^{N}\frac{\varphi_{i}}{SE\varphi_{i}}$$

(15) $$G_{a}=\frac{1}{N}\sum_{i-1}^{N}\frac{{T\varphi }_{i}}{\varphi_{i}\left(1\right)}$$

(16) $$P_{T}=\frac{\varphi_{i}}{SE\varphi_{i}}$$

(17) $$P_{a}={T\varphi }_{i}$$

#### 2.2.4 CS-ARDL

Cross-sectional auto-regressive distributed lagged (CS-ARDL) is a statistical technique used for analyzing the long-run relationship between a dependent variable and one or more independent variables in a cross-sectional setting. This technique combines the features of cross-sectional data and time-series data analysis to examine the long-term relationship between variables, considering both the time-series dynamics and cross-sectional heterogeneity [78,79]. In the CS-ARDL model, the dependent variable is regressed on its own lagged values, the independent variables' lagged values, the independent variables, and the cross-sectional averages of the independent variables. The model allows for different degrees of persistence across the individual units and heterogeneous coefficients for the independent variables across the cross-sectional units. CS-ARDL is particularly useful when dealing with cross-sectional data that exhibit heterogeneity and non-stationarity, as it allows for the inclusion of both time-varying and time-invariant variables in the analysis. The CS-ARDL model is commonly used in applied economics and finance to analyze the long-term relationships between economic growth, investment, and trade. It is also used in other fields, such as environmental studies, to examine the relationship between environmental quality and economic development across different countries.

Chudik and Pesaran [80] propose implementing Pesaran [75] Common Correlated Effects (CCE) approach in the context of panel ARDL models. Pesaran [75] displays the average values used in the Equation to represent unobserved common factors as a proxy of dependent and independent variables. Therefore, when averaging equations (16) and (17) across *time,* we obtain

(18) $${\bar{RE}}_{it}={\bar{\alpha }}_{it}+\sum_{j=1}^{p}{\bar{\beta }}_{ij}{\bar{RE}}_{i,t-j}+\sum_{j=0}^{q}{\bar{\gamma }}_{ij}{\bar{U}}_{i,t-j}+{\bar{\omega }}_{t}^{\prime }K_{t}+{\bar{\epsilon }}_{it}$$

Where, ${\bar{\alpha }}_{it}=\frac{\sum_{i-1}^{N}\alpha_{i}}{N}$.

$${\bar{RE}}_{t-j}=\frac{\sum_{i}^{N}{RE}_{i,t-j}}{N},{\bar{\beta }}_{j}=\frac{\sum_{i}^{N}\beta_{i,j}}{N}\;j=0,1,2\;p$$

$${\bar{U}}_{t-j}=\frac{\sum_{i}^{N}U_{i,t-j}}{N},{\bar{Ὑ}}_{j}=\frac{\sum_{i}^{N}{Ὑ}_{i,j}}{N},J=0,1,2\;q$$

$${\bar{\bar{\omega }}}_{j}=\frac{\sum_{i=1}^{N}\omega_{i}}{N},{\bar{\varepsilon }}_{t}=\frac{\sum_{i}^{N}\epsilon_{i,t}}{N}$$

The error term, ***ε****i*, in Eq. (6) is independently distributed across time and countries, and the mean congregates to zero (i.e., ε̅t = 0) in root mean square error as N → ∞. Therefore, the linear effects of both dependent and independents can be established in the presence of cross-sectional dependence in ***μ****i*,

(19) RE=α‾it+∑j=1pβ‾ijRE‾i,t−j+∑j=0qγ‾ijU‾i,t−j+ω‾t′Kt↓ω‾t′Kt=RE‾it−α‾it+∑j=1pβ‾ijRE‾i,t−j+∑j=0qγ‾ijU‾i,t−j↓Kt=RE‾it−α‾it+∑j=1pβ‾ijRE‾i,t−j+∑j=0qγ‾ijU‾i,t−jω‾t′

thus, the Panel CS-ARDL specification of Equation (2)

(20) $${\bar{RE}}_{it}=\epsilon_{it}+\sum_{j=1}^{p}\beta_{ij}{\bar{RE}}_{i,t-j}+\sum_{j=0}^{q}\gamma_{ij}{\bar{U}}_{i,t-j}+\sum_{j=0}^{p}{\bar{\partial }}_{tj}^{\prime }{\bar{K}}_{i,t-j}+\epsilon_{it}$$

Where $\bar{K}=\left(\bar{EE},\bar{EI,\bar{IQ,}}\right)$ and $S_{\bar{Z}}$ In the number of lagged cross-sectional averages furthermore, Equation (24) can be reparametrized to the effects of ECM presentation of Panel CS-ARDL as follows:

(21) $${\Delta RE}_{it}=\alpha_{i}+\xi_{i}\left({RE}_{it-1}-\omega_{t}^{\prime }U_{it-1}\right)+\sum_{J=1}^{M-1}\gamma_{iJ}{\Delta RE}_{it-J}+\sum_{J=0}^{N-1}\beta_{ij}{\Delta U}_{it-J}+\sum_{j=1}^{p}\lambda_{j}{\bar{\Delta RE}}_{i,t-j}+\sum_{j=0}^{q}\delta_{j}{\bar{\Delta U}}_{i,t-j}+\sum_{j=0}^{S_{\bar{Z}}}{\bar{\partial }}_{tj}^{\prime }{\bar{K}}_{i,t-j}+\mu_{it}$$

Where ${\bar{\Delta ES}}_{t-j}=\frac{\sum_{i}^{N}{\Delta ES}_{i,t-j}}{N}$, ${\bar{\Delta Q}}_{t-j}=\frac{\sum_{i}^{N}{\Delta Q}_{i,t-j}}{N}$.

#### 2.2.5 Nonlinear -ARDL

By following Shin, Yu [81], the following asymmetric model is to be tested:

(22) $${\Delta REC}_{it}=\beta_{0i}+\beta_{1i}REC+\beta_{2i}^{+}{EPU}_{t-1}^{+}+\beta_{2i}^{-}{EPU}_{t-1}^{-}+\beta_{3t}^{+}{DEBT}_{t-1}^{+}+\beta_{3t}^{-}{DEBT}_{t-1}^{-}+\beta_{4t}^{+}{FDI}_{t-1}^{+}+\beta_{4t}^{-}{FDI}_{t-1}^{-}+{\sum_{J=1}^{M-1}\gamma_{iJ}{\Delta REC}_{i,t-J}+\sum_{J=0}^{N-1}\left(\gamma_{ij}^{+}{\Delta EPU}_{i,t-j}^{+}+\gamma_{ij}^{-}{\Delta EPU}_{i,t-j}^{-}\right)+\sum_{J=0}^{O-1}\left(\left(\delta_{ij}^{+}{\Delta DEBT}_{i,t-j}^{+}+\delta_{ij}^{-}{\Delta DEBT}_{i,t-j}^{-}\right)\right)+\sum_{J=0}^{O-1}\left(\left(\Omega_{ij}^{+}{\Delta FDI}_{i,t-j}^{+}+\Omega_{ij}^{-}{\Delta FDI}_{i,t-j}^{-}\right)\right)+\varepsilon }_{it}$$

Where ${EPU}^{+}$& ${EPU}^{-}$ Stand for the positive and negative shock of economic policy uncertainty, ${FDI}^{+}$& ${FDI}^{–}$ represents the positive and negative shock of foreign direct investment and ${DEBT}^{+}$& ${DEBT}^{–}$ The positive and negative shock of government debt. The long-run coefficients are computed as ${Ὑ}^{+}=\frac{-\beta_{2i}^{+}}{\beta_{1i}}$, ${Ὑ}^{-}=\frac{-\beta_{2i}^{-}}{\beta_{1i}}$, $\mu^{+}=\frac{-\beta_{3i}^{+}}{\beta_{1i}}$, $\mu^{-}=\frac{-\beta_{3i}^{-}}{\beta_{1i}}$ , $\beta^{+}=\frac{-\beta_{3i}^{+}}{\beta_{1i}}$, ${and\;\beta }^{-}=\frac{-\beta_{3i}^{-}}{\beta_{1i}}\text{,}$ respectively. These shocks are computed as positive and negative partial sum decomposition of EPU, FDI, and DEBT in the following ways:

(23) $$\left\{ \begin{array}{c} {DEBT}_{i}^{+}=\sum_{k=1}^{t}{\Delta DEBT}_{ik}^{+}=\sum_{K=1}^{T}\mathrm{MAX}\left({\Delta DEBT}_{ik},0\right)\\ {DEBT}_{i}^{-}=\sum_{k=1}^{t}{\Delta DEBT}_{ik}^{-}=\sum_{K=1}^{T}\mathrm{MIN}\left({\Delta DEBT}_{ik},0\right) \end{array}\right.$$

(24) $$\left\{ \begin{array}{c} {FDI}_{i}^{+}=\sum_{k=1}^{t}{\Delta FDI}_{ik}^{+}=\sum_{K=1}^{T}\mathrm{MAX}\left({\Delta FDI}_{ik},0\right)\\ {FDI}_{i}^{-}=\sum_{k=1}^{t}{\Delta FDI}_{ik}^{-}=\sum_{K=1}^{T}\mathrm{MIN}\left({\Delta FDI}_{ik},0\right) \end{array}\right.$$

The error correction version of equation (10) is as follows:

(25) $${\Delta REC}_{it}=\tau_{1i}\xi_{it-1}+{\sum_{J=1}^{M-1}\gamma_{iJ}{\Delta REC}_{i,t-J}+\sum_{J=0}^{N-1}\left(\gamma_{ij}^{+}{\Delta EPU}_{i,t-j}^{+}+\gamma_{ij}^{-}{\Delta EPU}_{i,t-j}^{-}\right)+\sum_{J=0}^{O-1}\left(\left(\delta_{ij}^{+}{\Delta FDI}_{i,t-j}^{+}+\delta_{ij}^{-}{\Delta FDI}_{i,t-j}^{-}\right)\right)+\sum_{J=0}^{O-1}\left(\left(\Omega_{ij}^{+}{\Delta DEBT}_{i,t-j}^{+}+\Omega_{ij}^{-}{\Delta DEBT}_{i,t-j}^{-}\right)\right)+\varepsilon }_{it}$$

### 2.3 Dumitrescu-hurlin panel causality test

The study implements the granger causality test following the procedure initiated by The average Wald statistic of Dumitrescu and Hurlin's (https://doi.org/10.1016/j.econmod.2012.02.014 ">2012) panel causality test:

(26) $$Y_{it}=\alpha_{i}+\sum_{K-1}^{P}\gamma_{ik}Y_{i,t-k}+\sum_{K-1}^{P}\beta_{ik}X_{i,t-k}+\mu_{it}$$

(27) $$W_{NT}^{Hnc}=N^{-1}\sum_{i-1}^{N}W_{i,t}$$

(28) $$Z=\sqrt{\frac{N}{2P}\times \frac{T-2P-5}{T-P-3}}\times \left[\frac{T-2P-3}{T-2P-1}\bar{W}-P\right]$$

## 3 Estimation and interpretation

### 3.1 Cross-sectional dependency, heterogeneity test, panel unit root test, and panel cointegration test

The study implemented CSD tests with the null hypothesis of cross-sectional independence. The results of CSD tests are displayed in Table 2 and it is revealed that all the test statistics are statistically significant at a 1 % level. It advocates the cross-sectional dependency among the selected variables. Furthermore, the test statistics from the heterogeneity test were statistically significant, indicating the rejection of the null hypothesis of homogeneity.

**Table 2:** Reslts of cross-sectional dependency and heterogeneity test.

	${LM}_{BP}$	${LM}_{PS}$	${LM}_{adj}$	${CD}_{PS}$	Δ	Adj.Δ
lnREC	325.35***	32.966***	207.994***	7.979***	50.246***	56.454***
lnGI	182.513***	25.8***	191.519***	43.248***	64.937***	75.073***
lnTI	155.647***	45.415***	208.282***	33.022***	79.879***	142.281***
lnFO	441.877***	34.732***	186.308***	50.312***	59.002***	114.395***
lnGLO	443.938***	34.955***	209.508***	18.801***	61.916***	57.51***
lnUR	363.738***	35.726***	103.127***	6.99***	70.318***	109.836***
*lnFD*	330.043***	42.067***	148.918***	6.951***	32.392***	64.951***

The study assesses the variable's static properties by implementing the first-generation panel unit root tests following Levin, Lin [82], and Im, Pesaran [83], displayed in Table 3. Inferring to the test statistics, it is evident that all the test statistics are found statistically significant at a 1 % level after the first difference estimation (see Table 4).

**Table 3:** Results of First generation panel unit root test.

	Levin, Lin & Chu t		Im, Pesaran and Shin W-stat		ADF - Fisher Chi-square	
	t	t&c	t	t&c	t	t&c
*Panel –A: Al level*						
lnREC	−1.734	−2.261	−0.605	−3.393	42.307	39.29
lnGI	−3.087	−1.946	−1.287	−0.236	38.102	37.419
lnTI	−2.339	−2.431	−3.038	−3.965	58.806	52.054
lnFO	−2.714	−1.929	−0.037	−1.124	52.078	49.48
lnGLO	−1.261	−0.27	−1.267	−2.59	37.508	33.879
lnUR	−3.055	−0.391	−3.988	−0.094	42.639	50.193
*lnFD*	−2.694	−1.172	−3.181	−3.048	39.329	32.677
*Panel –B: After the first difference*						
lnREC	−9.051***	−18.63***	−7.898***	−9.023***	141.801***	164.223***
lnGI	−11.962***	−8.782***	−17.979***	−9.179***	168.685***	94.522***
lnTI	−6.132***	−16.757***	−7.638***	−9.425***	127.041***	134.234***
lnFO	−9.976***	−19.57***	−10.645***	−8.114***	290.803***	94.982***
lnGLO	−7.547***	−17.749***	−5.776***	−8.754***	275.978***	139.48***
lnUR	−7.901***	−17.685***	−20.676***	−7.357***	236.091***	131.283***
*lnFD*	−9.359***	−20.71***	−8.546***	−9.228***	161.819***	188.131***

**Table 4:** Results of Second generation unit root test.

	CIPS		CADF	
	At level	Δ	At level	Δ
lnREC	−2.997	−4.774***	−1.994	−3.911***
lnGI	−2.339	−6.381***	−1.816	−3.714***
lnTI	−2.858	−5.645***	−1.076	−3.42***
lnFO	−1.817	−2.424***	−2.081	−6.377***
lnGLO	−2.819	−2.842***	−1.897	−3.398***
lnUR	−1.359	−6.62***	−1.595	−2.999***
*lnFD*	−2.925	−2.833***	−1.791	−2.047***

Considering the CSD test results, the study performed second-generation panel unit root tests commonly known as CIPS and CADF, which are familiar to Pesaran [84]. Mentioning the test statistics derived from CIPS and CADF, the study revealed that all the variables are stationary after the first difference operation, which is considered for selecting and implementing the appropriate and advanced economic techniques to assess the empirical nexus.

The long-run association between explained and explanatory variables has been gauged through the execution of a panel cointegration test following Pedroni [85] and Westerlund [86]. The results of the panel cointegration test are reported in Table 5. Indicating the test statistics derived from the conventional panel cointegration test, 9 out of 11 test statistics have divulged statistical significance at a 1 % level, suggesting the long-run association in the Equation. Likewise, the results derived from the error correction term study established that all four coefficients are statistically significant at a 1 % level. The conclusive evidence from both estimations confirmed the long-run cointegration between RC, GI, TI, and FO. Now, we proceed to explore the long-run and short-run coefficients by implementing CS-ARDL, which was introduced by Chudik and Pesaran [80].

**Table 5:** Results of Panel cointegration test.

Panel A: Pedroni Cointegration			
Panel v-Statistic	Panel rho-Statistic	Panel PP-Statistic	Panel ADF-Statistic
1.319	−4.347	−8.348	−3.357
Panel v-Statistic	Panel rho-Statistic	Panel PP-Statistic	Panel ADF-Statistic
−0.82	−6.297	−9.02	−10.533
Group rho-Statistic	Group PP-Statistic	Group ADF-Statistic	
−9.733	−6.685	−4.877	
Panel B: Error correction Based Cointegration			
Gt	Ga	Pt	Pa
−11.141***	−10.936***	−14.304***	−12.823***

### 3.2 Baseline assessment: OLS, random effects, and fixed effects

Referring to Hausman test statistics, the fixed-effects estimation is efficient and consistent. Considering the empirical model estimation output with FE, the study revealed a positive statistically significant linkage between GI and REC, suggesting the beneficiary role of GI for the development of green energy in the form of renewable sources. Referring to the coefficients of technological innovation, it is apparat that technological advancement in the economy positively augments clean energy consumption through the inclusion of renewable energy sources. Technological innovation prompts energy transition from fossil fuel to renewable sources and ensures energy efficiency. Financial openness revealed a catalyst for RE development in the economy, implying that the inflows of FDI hasten energy growth, especially targeting renewable sources. Regarding control variables' influences on REC, the study disclosed a positive statistically significant linkage between control variables: urbanization, globalization, financial development, and REC Table 6.

**Table 6:** Results of baseline investigation.

	OLS	RF	FE
lnGI	0.208(0.0332)[-6.249]	0.567(0.0455)[12.453]	0.196(0.0203)[9.631]
lnTI	−0.067(0.0127)[-5.236]	0.221(0.0369)[5.981]	0.607(0.0481)[12.618]
lnFO	0.407(0.033)[12.33]	0.679(0.1452)[4.675]	0.457(0.066)[6.918]
lnGLO	0.712(0.0884)[8.047]	0.166(0.0214)[7.739]	0.314(0.0413)[7.59]
lnUR	0.161(0.0132)[12.188]	0.498(0.0577)[8.628]	−0.162(0.0278)[-5.82]
*lnFD*	0.576(0.0503)[11.445]	−0.167(0.039)[-4.274]	0.286(0.0306)[9.343]
constant	−0.161(0.0338)[-4.763]	0.354(0.0877)[4.035]	0.27(0.0239)[11.266]
H-test			

### 3.3 Impact of green investment, technological innovation, financial openness, and renewable energy consumption: CS-ARDL estimation

The coefficients of green investment (GI) revealed a positive statistically significant connection to renewable energy consumption (REC) both in the long-run (a coefficient of 0.1742) and short-run (a coefficient of 0.0404), suggesting the contributory role of GI on the argumentation of REC in the economy. More precisely, a 10 % growth GI in the economy will increase REC by 1.742 % in the long-run assessment and 0.404 % in the short-run. The positive tie between GI and REC is in line with existing literature, such as [87], Inferring the nexus between technological innovation and renewable energy accessibility, the study documented a positive statistically significant linkage between them in the long-run and short-run valuation. More precisely, a 1 % growth in technological innovation can intensify the process of renewable energy presence by increasing the share of total energy consumption by 0.1344 % in the long run and by 0.0364 % in the short run. Study findings suggest that the progress in TI positively augments clean energy inclusion. Our findings align with the existing literature [88]. Nonetheless, a contrasting view is posed by the study of [59] in BRI countries.

The coefficients of FO are positively associated with REC in the long run (a coefficient of 0.152) and short-run (a coefficient of 0.0418), which is statistically significant at a 1 % level. According to FO elasticity, in the long run (short-run), energy consumption will increase by 0.152 % (0.0418 %) due to a 1 % innovation in FO. Our study findings postulate that foreign ownership presence in the economy prompts RE demand compared to conventional energy, suggesting that FDI inflows bring technological advancement, ensuring operational efficiency, especially in the long run. Thus, it is anticipated that the demand for clean energy will possibly grow. The existing literature supports our study findings. See Refs. [31,63,89,90].

Considering the effects of control variables, the study establishes that urbanization (a coefficient of 0.1381), globalization (a coefficient of 0.1545), and financial development (a coefficient of 0.1504) are positively augmenting energy development, specifically through renewable sources, in the long run. Meanwhile, the coefficients of short-run assessment disclose positive associating between globalization (a coefficient of0.0239), financial development (a coefficient of 0.0373), and renewable energy consumption, whereas adverse association documents between urbanization (a coefficient of of-0.0545) and renewable energy consumption of.

### 3.4 Asymmetric nexus between GI, TI, FO, and RE

Next, the present study has implemented the nonlinear framework following shin in exploding the asymmetric influences of green investment (${GI}^{+},{GI}^{-}$), technological innovation (${TI}^{+},{TI}^{-}$), and financial openness (${FO}^{+},{FO}^{-})$ (see Table 7). The asymmetric assessment results are displayed in Table 8.

**Table 7:** Long-run and short-run coefficients: CS-ARDL.

	Coefficient	t-stat	std. Error		Coefficient	t-stat	std. Error
***Long-run coefficients***				***Short-run coefficients***			
**GI**	0.0472	0.0067	7.0447	**ΔGI**	0.0404	0.0105	3.8476
TI	0.1344	0.0121	6.4110	ΔTI	0.0364	0.0075	4.8533
F0	0.152	0.0049	31.0204	ΔF0	0.0418	0.0064	6.5312
UR	0.1381	0.0031	44.5483	ΔUR	−0.0545	0.0052	−10.4807
GLO	0.1545	0.0049	31.5306	ΔGLO	0.0239	0.0116	2.0603
FD	0.1504	0.0021	71.619	ΔFD	0.0373	0.0091	4.0989
Constant	−0.1158	0.0064	−18.0937	ECT(-1)	−0.1725	0.0054	−31.9444
Diagnostic test							
CD statistics				12.841***			
WT for autocorrelation				0.214			
Normality test				3.412			
Ramsey RESET test				1.151

**Table 8:** Asymmetric long-run and short-run coefficients.

							
	Long-run coefficients				Short-run coefficients		
GI_NEG	0.0768	0.02343	3.2778	ΔGI_NEG	0.0558	0.04068	1.3716
GI_POS	0.1143	0.01869	6.1155	ΔGI_POS	0.0756	0.01967	3.8434
TI_POS	0.1107	0.04231	2.6164	▲TI_POS	0.0764	0.03725	2.051
TI_NEG	0.067	0.03162	2.1189	▲TI_NEG	0.0849	0.04063	2.0895
FO_POS	0.1221	0.01061	11.508	▲FO_POS	0.0338	0.02586	1.307
FO_NEG	0.0664	0.03592	1.8485	▲FO_NEG	0.0452	0.03905	1.1574
UR	0.0997	0.03451	2.889	▲UR	0.0748	0.02177	3.4359
GLO	0.077	0.045	1.7111	▲GLO	0.0265	0.0207	1.2801
FD	0.109	0.04896	2.2263	▲FD	0.0604	0.04132	1.4617
C	10.51053	0.00898	1170.4376	ECT (−1)	−0.34178	0.02828	−12.0855
Test symmetry							
$W_{LR}^{GI}$		15.2984***		$W_{SR}^{GI}$		5.0811***	
$W_{LR}^{TI}$		13.7771***		$W_{SR}^{TI}$		3.2986***	
$W_{LR}^{FO}$		14.0157***		$W_{SR}^{FO}$		2.0185**	
CD statistics				9.8451***			
WT for autocorrelation				0.1551			
Normality test				2.612			
Ramsey RESET test				2.6112			

In terms of the standard Wald test statistics for investigating the long-run and short-run asymmetric association between an explained variable and explanatory variables. The study unveiled the Wald test statistics of $W_{LR}^{GI}=15.2984$, $W_{LR}^{TI}=13.7771$, and $W_{LR}^{FO}=14.0157$ statistically significant at a 1 % level, indicating the long-run asymmetric linkage. Furthermore, the Wald test statistics for short-run asymmetry is $W_{SR}^{GI}=5.0811$, $W_{SR}^{TI}=3.2986$, and $W_{SR}^{FO}=2.0185$, has exposed the statistically significant and confirmed the asymmetric tie between explained and expletory variables.

Inferring to the asymmetric coefficients of GI that are positive and negative shocks, the study revealed a positive statistically significant tie with renewable energy in the long-run and short-run assessment, suggesting the positive (negative) variations in green investment have acute effects on renewable energy growth in the economy. More precisely, a 1 % variation in GI can cause renewable energy to progress by 0.0768 % in the long run and by 0.0558 % in the short run. At the same time, the negative innovation in GI can dwindle the clean energy inclusion through renewable sources by 0.1143 % in the long run and by 0.0756 % in the short run. The long- and short-run assessment documented a positive and statistically significant link between asymmetric technological innovation shocks and REC. In terms of long-run estimation, due to a 10 % positive (negative) change in TI has exemplified (degraded) the REC in the MINT economy by 1.107 %(0.671 %). In contrast, in the short-run, the overall REC has increased (decreased) by 0.0764 % (0.0849 %) in the case of a 1 % progress (degrees) in TI. TI has a deterministic influence on clean energy inclusion by promoting renewable energy sources in the long and short run.

The asymmetric effects of financial openness on REC have exposed positive statistical significance in the long and short-run horizon. In particular, a 1 % positive (negative) variation in FO will result in acceleration (degradation) of REC in MINT by 0.1221 % (0.0664 %). Moreover, the REC in the sample economy will increase (decrease) by 0.0338 % (0.0452 %) due to a 1 % positive (negative) innovation in FO. Study findings advocate that FO offers technological transitions in the host economy along with energy efficiency; precisely, FO ignites the movements in clean energy development and inclusion in the industrial process.

### 3.5 Directional causality: linear and nonlinear effects of explanatory variables

Next, the present study implemented the casualty assessment following the concept familiar to Dumitrescu and Hurlin [4]. The results are reported in Table 9. Bidirectional causality revealed between green investment and renewable energy (GI←→RE); globalization and renewable energy [GLO←→RE]; financial development and renewable energy [FD←→RE]. Furthermore, the unidirectional causality documented between renewable energy consumption and financial openness [REC→FO], technological innovation, and renewable energy consumption [TI→REC].

**Table 9:** Results of panel causality test.

	RE	GI	TI	FO	UR	GLO	FD
RE	–	(4.2369)** [4.4657]	(5.2784)*** [5.5634]	0.8852 [0.933]	1.2911 [1.3609]	(5.2816)*** [5.5668]	(5.306)*** [5.5925]
GI	(3.1424)** [3.312]	–	1.3156 [1.3866]	(2.458)* [2.5907]	(2.2763)* [2.3992]	(5.1211)*** [5.3976]	(4.6142)** [4.8634]
TI	1.424 [1.5009]	(4.4027)** [4.6405]	–	1.2954 [1.3653]	(5.5302)*** [5.8289]	(4.8129)*** [5.0728]	(4.0967)** [4.3179]
FO	(5.2869)*** [5.5724]	(2.1625)* [2.2793]	(2.3772)* [2.5056]	–	(3.8533)** [4.0614]	1.5759 [1.661]	(2.5579)* [2.696]
UT	1.154 [1.2164]	1.4144 [1.4908]	(4.8575)*** [5.1199]	1.0818 [1.1402]	–	1.7151 [1.8078]	1.6344 [1.7226]
GLO	(4.8544)*** [5.1165]	(2.4909)* [2.6254]	(5.0138)*** [5.2845]	(5.8831)*** [6.2007]	(4.6928)** [4.9462]	–	(4.1126)** [4.3347]
FD	(5.0499)*** [5.3226]	(3.4165)** I[3.601]	(5.9096)*** [6.2287]	(2.7885)* [2.9391]	(6.0042)*** [6.3284]	1.4218 [1.4986]	–

The directional causality has been extended by incorporating explanatory variables' asymmetric shocks on REC (see Table 10). Table 11 displays the results of the causality test with asymmetric explanatory variables. Taking into account the test statistics, it is apparent that the feedback hypothesis holds in elucidates the causality between renewable energy consumption and positive changes in green investment [REC←→ ${GI}^{+}$], and financial openness [REC←→ ${FO}^{+}$].

**Table 10:** D-H Results of asymmetric causality.

Null Hypothesis:	W-Stat.	Zbar-Stat.		W-Stat.	Zbar-Stat.	
FO measures by Carbon emission				FO measures by ecological footprint		
${GI}^{+}$ ≠→REC	5.9851	6.3083***	REC←→ ${GI}^{+}$	3.4835	3.6716*	${GI}^{+}$ →REC
REC≠→ ${GI}^{+}$	4.4962	4.7390**		4.8480	5.1098***	
${GI}^{-}$ ≠→REC	3.1987	3.3714*	REC← ${GI}^{-}$	3.6004	3.7948*	REC← ${GI}^{-}$
REC≠→ ${GI}^{-}$	2.0807	2.1931		0.8331	0.8781	
${TI}^{+}$ ≠→REC	4.0765	4.2966**	REC← ${TI}^{+}$	4.3103	4.5435**	REC← ${TI}^{+}$
REC≠→ ${TI}^{+}$	1.1562	1.2186		0.9149	0.9643	
${TI}^{-}$ ≠→REC	3.9617	4.1756**	REC← ${TI}^{-}$	5.10627	5.3829***	REC← ${TI}^{-}$
REC≠→ ${TI}^{-}$	2.0329	2.1427		2.2709	2.3936	
${FO}^{*+}$ ≠→REC	4.2295	4.4579**	REC←→ ${FO}^{+}$	6.1381	6.4696***	${FO}^{+}-\to REC$
REC≠→ ${FO}^{*+}$	3.6843	3.8833*		3.9309	4.1431	
${FO}^{-}$ ≠→REC	1.6939	1.7854	REC← ${FO}^{-}$	5.9107	6.2299***	REC←→ ${FO}^{-}$
REC≠→ ${FO}^{-}$	5.6726	5.9790***		5.4229	5.7157***

**Table 11:** Results of the coefficients robustness test.

	Coefficient	t-stat	std. Error	Coefficient	t-stat	std. Error	Coefficient	t-stat	std. Error
	MG			AMG			CCEMG		
GI	0.0737	0.0024	30.7083	0.0839	0.0081	10.358	0.0231	0.0051	4.5294
TI	0.0683	0.0038	17.9736	0.0922	0.0025	36.88	0.0205	0.0056	3.6607
FO	0.0656	0.0093	7.0537	0.0535	0.0116	4.612	0.015	0.0033	4.5454
UT	0.064	0.0085	7.5294	0.0621	0.0094	6.6063	0.0757	0.0037	20.4594
GLO	0.0707	0.01	7.07	0.0758	0.0043	17.6279	0.0421	0.0114	3.6929
FD	0.0156	0.0107	1.4579	0.0724	0.0077	9.4025	0.0111	0.0099	1.1212
	0.0735	0.009	8.1666	0.0163	0.0093	1.7526	0.0312	0.0108	2.8888
Wald test		0.0032			0.0085			0.0062	
CD test		0.0108			0.0099			0.008	

### 3.6 Further discussion

Next, the empirical Equations (1), (2) have been implemented following the framework proposed by Eberhardt [3], commonly known as AMG and Correlated Effect Mean Group (CCEMG) by Pesaran [75](2006) methods for confirming the robustness in the long-run coefficient. The results of the robustness test are displayed in Table 11. Referring to the sign of explanatory variables coefficients on an explained variable, it is apparent that all the coefficients have exposed a similar vine of association established in prior estimation.

### 3.7 Country-wise assessment

#### 3.7.1 Long-run run and short-run coefficients of ARDL estimation

Next, the study has extended the empirical assessment by executing the target model by taking account of country-specific assessment. The results of ARDL are displayed in Table 12, consisting of a four-panel output. The test statistics for long-run cointegration are statistically significant at a 1 % level, confirming the long-run association between explained and explanatory variables.

**Table 12:** Results of ARDL model estimation.

	Morocco	Indonesia	Turkey	Nigeria
**Panel-A: long-run cointegration**				
F_*overall*_	8.199***	9.325***	13.197***	10.747***
t_DV_	−5.807***	−6.095***	−6.905***	−5.773***
F_*IDV*_	8.51***	10.078***	9.46***	6.301***
**Panel –B: Long-run coefficients**				
GI	0.069(0.0053) [13.0188]	0.1624(0.0085) [19.1058]	0.1624(0.0085) [19.1058]	0.1302(0.0034) [38.2941]
TI	0.0657(0.0035) [18.7714]	0.0991(0.0044) [22.5227]	0.0991(0.0044) [22.5227]	0.0354(0.011) [3.2181]
FO	0.1436(0.0088) [16.3181]	0.1258(0.0029) [43.3793]	0.1258(0.0029) [43.3793]	0.0339(0.0043) [7.8837]
UT	0.0417(0.011) [3.7909]	0.0739(0.0026) [28.423]	0.0739(0.0026) [28.423]	0.1254(0.0113) [11.0973]
GLO	−0.1749(0.0079) [-22.1392]	−0.0547(0.0067) [-8.1641]	−0.0547(0.0067) [-8.1641]	−0.0744(0.0107) [-6.9532]
FD	1.5501(0.533) [2.9082]	3.1872(0.4512) [7.0638]	3.1872(0.4512) [7.0638]	3.3712(7.9084) [0.4262]
**Panel –C: Short-run coefficients**				
GI	0.0982(0.0053) [18.5283]	0.076(0.0092) [8.2608]	0.076(0.0092) [8.2608]	0.0367(0.0103) [3.5631]
TI	0.0346(0.0099) [3.4949]	0.0717(0.0034) [21.0882]	0.0717(0.0034) [21.0882]	0.0405(0.0069) [5.8695]
FO	−0.0471(0.002) [-23.55]	−0.0514(0.0099) [-5.1919]	−0.0514(0.0099) [-5.1919]	−0.0863(0.0086) [-10.0348]
UT	−0.1023(0.011) [-9.3]	−0.068(0.0087) [-7.816]	−0.068(0.0087) [-7.816]	−0.0896(0.007) [-12.8]
GLO	0.1075(0.0082) [13.1097]	0.0746(0.0101) [7.3861]	0.0746(0.0101)[7.3861]	0.1053(0.0035) [30.0857]
FD	−0.3021(0.0085)[-35.5495]	−0.2633(0.0812) [-3.2429]	−0.2633(0.0812)[-3.2429]	−0.4748(0.0862) [-5.5091]
ECT(-1)				
**Panel –D: Residual Diagnostic test**				
$x_{Auto}^{2}$	0.656	0.633	0.62	0.689
$x_{Het}^{2}$	0.715	0.542	0.796	0.872
$x_{Nor}^{2}$	0.645	0.632	0.698	0.707
$x_{RESET}^{2}$	0.588	0.826	0.839	0.645

The long-run output in the panel study displayed a positive statistically significant tie between renewable energy consumption and target explanatory variables such as green investment, technological innovation, and financial openness. The conclusion of the positive connection between explained and explanatory variables is confirmed for the selected four countries. Moreover, regarding short-run assessment (see Panel –C), green investment and technological innovation coefficients disclosed positively associated with renewable energy consumption. Nonetheless, a negative association was revealed between financial openness and renewable energy consumption. The residual diagnostic test results stand for model construction and estimation efficiency.

#### 3.7.2 Long-run run and short-run coefficients of nonlinear ARDL estimation

In the following, the asymmetric investigation is executed to check the possible asymmetric linkage between asymmetric shocks of explanatory variables and renewable energy consumption. Table 13 exhibited asymmetric estimation results, including the asymmetric cointegration results in Panel –A, long-run coefficients in Panel –B, Panel –C deals with the short-run coefficients, and the residual diagnostic test reported in Pane –D, respectively (see Table 12).

**Table 13:** Results of long-run run a*nd short-run coefficients of nonlinear ARDL estimation*.

	Morocco	Indonesia	Turkey	Nigeria
Panel –A: asymmetric long-run cointegration				
F_*overall*_	12.575***	7.455***	11.791***	9.281***
t_DV_	−5.83***	−6.916***	−5.95***	−6.686***
F_*IDV*_	11.238***	10.436***	7.774***	11.188***
Panel –B: asymmetric long-run coefficients				
GI_NEG	0.0311(0.0108) [2.871]	0.0427(0.0089) [4.7893]	0.1482(0.0072) [20.5426]	0.0299(0.0078) [3.82]
GI_POS	0.017(0.008) [2.1304]	0.075(0.004) [18.4925]	0.0333(0.0113) [2.9409]	0.1853(0.0039) [47.1582]
TI_POS	0.1229(0.0112) [10.8985]	0.0335(0.0041) [8.0568]	0.0594(0.0019) [31.3082]	0.03(0.0088) [3.381]
TI_NEG	0.0864(0.0072) [11.8573]	0.07(0.0032) [21.713]	0.1217(0.0029) [41.2566]	0.1281(0.0029) [43.3528]
FO_POS	0.0482(0.007) [6.8177]	0.0594(0.0036) [16.2949]	0.1403(0.0088) [15.8359]	0.1092(0.0116) [9.3488]
FO_NEG	0.1069(0.0035) [29.7229]	0.0406(0.0071) [5.7091]	0.0683(0.0103) [6.5925]	0.0521(0.0064) [8.1068]
UR	0.088(0.0117) [7.5122]	0.0533(0.0053) [10.0065]	0.0313(0.0028) [10.832]	0.0601(0.0057) [10.5164]
GLO	0.0651(0.005) [12.8325]	0.1103(0.0114) [9.6687]	0.0533(0.0099) [5.3755]	0.0184(0.0051) [3.5494]
FD	0.0297(0.0113) [2.6318]	0.0963(0.0075) [12.7561]	0.0578(0.0116) [4.9677]	0.1122(0.0107) [10.4661]
Panel –C: short-run asymmetric coefficients				
${\Delta GI}^{+}$	0.0253(0.0113) [2.2389]	0.0262(0.0042) [6.238]	0.0065(0.0036) [1.8055]	0.0176(0.0082) [2.1463]
${\Delta GI}^{-}$	0.0268(0.0043) [6.2325]	0.0351(0.0106) [3.3113]	0.0314(0.0021) [14.9523]	0.0459(0.0101) [4.5445]
${\Delta TI}^{+}$	0.0663(0.0054) [12.2777]	0.0541(0.0035) [15.4571]	0.0056(0.0056) [1.016]	0.0431(0.0068) [6.3382]
${\Delta TI}^{-}$	0.0426(0.0065) [6.5538]	0.0329(0.0053) [6.2075]	0.0474(0.0098) [4.8367]	0.0233(0.009) [2.5888]
${\Delta FO}^{+}$	0.0315(0.009) [3.5]	0.0422(0.0032) [13.1875]	0.0342(0.0033) [10.3636]	0.0363(0.0021) [17.2857]
${\Delta FO}^{-}$	0.0571(0.0071) [8.0422]	0.0433(0.0093) [4.6559]	0.0365(0.0106) [3.4433]	0.0669(0.0112) [5.9732]
ΔUR	0.053(0.0118) [4.4915]	0.0288(0.0096) [3]	0.0647(0.0091) [7.1098]	0.041(0.0068) [6.0294]
ΔGLO	0.066(0.0106) [6.2264]	0.033(0.0096) [3.4375]	0.0537(0.0046) [11.6739]	0.0307(0.0024) [12.7916]
ΔFD	0.1449(0.0044) [32.9443]	0.3301(0.0054) [60.3092]	0.0559(0.0041) [13.4572]	0.5963(0.0039) [151.8077]
ECT(-1)	−0.5522(0.1151) [-4.7976]	−0.3697(0.1021) [-3.6218]	−0.4156(0.1245) [-3.3381]	−0.4368(0.1023) [-4.2703]
Panel –D: symmetry test				
$W_{LR}^{GI}$	12.549	11.796	11.093	3.617
$W_{LR}^{TI}$	12.398	9.334	5.761	9.032
$W_{LR}^{FO}$	3.957	6.838	8.953	6.242
$W_{LR}^{GI}$	6.144	6.742	9.595	11.543
$W_{LR}^{TI}$	9.869	4.208	10.616	10.594
$W_{LR}^{FO}$	3.11	10.252	7.84	7.34
Panel –D: Residual Diagonestic test				
$x_{Auto}^{2}$	0.676	0.645	0.533	0.682
$x_{Het}^{2}$	0.615	0.82	0.633	0.72
$x_{Nor}^{2}$	0.51	0.541	0.611	0.718
$x_{RESET}^{2}$	0.844	0.566	0.733	0.782

Test statistics dealing with asymmetric cointegration were found statistically significant, suggesting the asymmetric association available between explanatory variables and explained variables. The asymmetric shocks of explanatory variables that are positive and negative shocks in GI, TI, and FO on renewable energy consumption have documented and exposed a positive and statistically significant association between them. Study findings suggest that the positive (negative) GI, TI, and FO variations will accelerate (contract) clean energy inclusion in industrial output. Alternatively, the study established the catalyst role of GI, TI, and FO in renewable energy development and energy transition from conventional to renewable energy sources.

## 4 Discussion

The study documented a positive tie between green investment and renewable energy under the assumption of a symmetric and asymmetric framework, suggesting that the growth of green investment accelerated clean energy sources. The progress of RE with the assistance of green investment was established in our study, which aligns with the existing literature [91,92]. More precisely, referring to the CS-ARDL estimation, a 10 % increase in GI results in the acceleration of renewable energy concentration in the total energy consumption by 1.742 % in the long run and 0.404 % in the short run. Furthermore, according to asymmetric assessment, the asymmetric shocks in GI, i.e., GI^+^ and GI^−^, have revealed a positive statistically significant linkage with RE in the long-run (GI^+^ = 0.0768; GI^−^ = 0.1143) and short-run (GI^+ =^0.0558; GI^−^ = 0.0756). In particular, a 10 % progress (degrees) in GI in the long run results in acceleration (degradation) of RE by 0.768 % (1.1143 %) in the long run, and for the short-run renewable energy influenced by 0.558 %(0.756 %). Regarding magnitudes of asymmetric shocks, it is apparent that the adverse shocks in GI have produced significant scratches on renewable energy development. This is valid in the long-run and short-run investigations. This is pustulating that construction policies focus theprivate–public investment on green projects, particularly renewable energy sources and environmental innovation. Green financing is an essential part of the agenda for green economic growth. Green finance refers to funding public and private sector investments that are good for the environment and the community. “green finance” refers to tools designed to assist companies in investing in environmentally friendly materials. Production, transportation, mining, and tourism are just a few industries that depend primarily on the constant availability of energy sources [70,93]. The growth of green finance has accelerated economic and operational performance through the assurance of economic resource optimization by lowering the cost of environmental protection with the input of renewable energies [94]. To promote the use of renewable resources for energy production, green finance enables companies to implement renewable energy initiatives [95].

In a nuts shell, Investments in ecologically responsible initiatives, such as renewable energy, are frequently what are meant by the term “green investment.” The effects of green investment in REC on the economy can be observed in the following manner. Due to the large initial financial commitment, it may be challenging for businesses or governments to fund renewable energy projects independently. Green investment is a way to fund the research, development, and widespread implementation of renewable energy initiatives. Second, green investment is key in expanding renewable energy because it provides financial incentives for new renewable energy projects and technologies. As a result, new businesses have emerged, such as the production of solar panels and wind turbines, which have the potential to boost the economy and create new jobs. Fourth, firms' efforts to attract Green investment by developing better and more efficient renewable energy technologies may spur innovation in that field. As a result, we have seen improvements in areas such as solar panel efficiency, wind turbine design, and battery technology. Forth, renewable energy sources are crucial in the fight against climate change and for greater environmental preservation. Reduced greenhouse gas emissions and less reliance on fossil fuels are two positive outcomes that may result from green investment in renewable energy projects. Since it uses naturally regenerated resources, renewable energy is a long-term, sustainable option for meeting our energy needs. Sustainable renewable energy projects have a better chance of being maintained with the aid of green investment, which helps to provide steady funding and support. Finally, Green investment offers the funding and assistance essential to propel the expansion and innovation of renewable energy projects, making the two sectors intrinsically linked. More money going into renewable energy sources means greater innovation and development opportunities in this crucial industry [96].

Both symmetric and asymmetric nexus between technological innovation and renewable energy consumption have exposed a positive and statistically significant linkage in the long and short run. It advocates the contributory role in clean energy development, including renewable energy. Our study findings are in line with the existing literature do Valle Costa, La Rovere [33], Jin, Duan [97], Sharma, Shahbaz [98], Khan, Su [32], Zhuo and Qamruzzaman [99]. Technological progress is crucial for energy progress and transition, implying that efficient energy production and replacement instead of conventional energy requires technological subsidy; that is the industry's scope to capitalize on the available technological assistance. Thus, growing researchers have advocated fund mobilization for innovation, especially for technology upgradation [67,100,101]. For the case of BRI, Khan, Chenggang [59] documented beneficial role of technological innovation on energy selection especially for the development of renewable energy sources.

Technological advancement has largely facilitated renewable energy's incorporation into the energy balance. Here are some ways in which technological innovation has facilitated the growth and integration of renewable energy: First, TI in renewable energy, such as enhancements in solar panel and wind turbine efficacy, have resulted in substantial decreases in the cost of producing renewable energy. This has made renewable energy more cost-competitive with conventional fossil fuels, increasing its appeal to investors and consumers. Second, TI has also led to advancements in energy storage technology, essential for ensuring that renewable energy can be integrated into the grid reliably. Energy storage systems, such as batteries and pumped hydro storage, enable renewable energy to be retained and utilized when required, as opposed to only when it is being generated. Third, TI has also resulted in enhancements to grid management systems, which are essential for integrating renewable energy into the grid. Sophisticated grid technologies, such as sensors and analytics tools, enable grid administrators to anticipate better and manage energy demand, making integrating renewable energy sources into the grid simpler. Fourth, TI has enabled the decentralization of energy production, permitting the development of smaller-scale renewable energy initiatives that can be located closer to the consumption site. This has made renewable energy accessible to various stakeholders, including individuals and small businesses. Technological innovation has played a crucial role in accelerating the incorporation of renewable energy into the energy balance by reducing costs, enhancing energy storage and grid management systems, facilitating decentralization, and fostering the development of hybrid energy systems. Renewable energy will probably continue to expand and be incorporated into the energy balance as technological innovation advances [102].

The association between financial openness and renewable energy consumption has been positively connected, suggesting that financial openness boosts renewable energy consumption and progress. The positive linkage between FO and REC is supported by the existing literature such as Qamruzzaman and Jianguo [62], Rasoulinezhad and Saboori [65], Koengkan, Fuinhas [63], Koengkan, Fuinhas [89]. Investments in renewable energy are encouraged by the maturation of the financial system, which reduces financing costs and eliminates adverse selection and moral hazard. This impact is magnified for more capital-intensive energy sources that rely on external financing. The study of Lin, Omoju [103] has documented that financial support is the key to renewable energy development, meaning that capital support from domestic or/and external sources has to be confirmed to the energy transition process from conventional to renewable sources might not dwindle. Thus, for financial support, the economic openness that is the scope to attract foreign investors in the energy sectors can be considered the best way to develop energy [18].

## 5 Conclusion and suggestions

Development and inclusion of renewable energy instead of fossil fuel have multidimensional effects on the economy, particularly in correcting environmental imbalance and economic sustainability by lowering the environmental protection cost. The study aims to assess the effects of green investment, technological innovation, and financial openness on renewable energy consumption in the MINT economy from 1990 to 2019. The study implemented several econometrical techniques in assessing the targeted nexus, such as the CDS test, PURT following CADF and CIPS [84], error correction-based panel cointegration test [86], CS-ARDL [80], Nonlinear ARDL [104] and D-H causality test. The key findings are as follows.

First, the slope of the homogeneity and cross-sectional dependency test results revealed that research units possessed heterogeneity properties and were cross-sectionally dependent. The results of the panel unit roots test, CADF, and CIPS disclosed that the variables become stationary after the first difference, suggesting the variable's order of integration after the first difference, i.e., I(1).

Second, the output from the pane cointegration test dealing with the determination of long-run association between explained and explanatory variables has revealed statistically significant at a 1 % level. This suggests a long-run relation in the empirical Equation.

Third, according to the coefficients derived from CS-ARDL estimation, GI, TI, and FO on REC have exposed positive and statistically significant at a 1 % level in the long-run and short-run assessment. This advocates the beneficial effects of GI, TI and FO in the clean energy transition from conventional to renewable sources. The nonlinear assessment revealed an asymmetric association both in the long-run and short-run horizon. Regarding the asymmetric coefficients of GI, TI, and FO on REC, the study unveiled a positive and statistically significant linkage, implying that any fluctuation in explanatory variables would result in REC movements.

Fourth, the directional casualty results in the feedback hypothesis hold between green investment and renewable energy (GI←→RE); globalization and renewable energy [GLO←→RE]; financial development and renewable energy [FD←→RE]. Furthermore, the unidirectional causality documented between renewable energy consumption and financial openness [REC→FO], technological innovation, and renewable energy consumption [TI→REC].

MINT nations (Mexico, Indonesia, Nigeria, and Turkey) are countries with rapidly growing economies and populations, which are also major emitters of greenhouse gases. Clean energy development is critical for these nations to meet their growing energy demand while reducing their carbon footprint. Here are some policy suggestions for promoting clean energy development in MINT nations through green investment, technological innovation, and financial openness.

1. MINT nations should develop policies to incentivize green investment, such as tax breaks or subsidies for renewable energy projects. This would help to attract more private investment in renewable energy infrastructure and technology, which is crucial for promoting the development and adoption of clean energy.
2. MINT nations should also invest in research and development of renewable energy technologies to reduce costs and increase efficiency. This can be achieved through funding for research and development programs or through public-private partnerships that leverage private sector expertise and resources.
3. MINT nations should promote financial openness and remove barriers to foreign investment in the renewable energy sector. This can be achieved by simplifying regulatory processes for foreign investors, reducing trade barriers, and ensuring the protection of foreign investments.
4. MINT nations should invest in renewable energy infrastructure, such as wind and solar farms, energy storage facilities, and smart grids, enabling the integration of renewable energy sources into the existing energy mix, reducing reliance on fossil fuels and promoting clean energy use.
5. MINT nations should establish public-private partnerships to promote clean energy development. Such partnerships would enable collaboration between governments, private sector companies, and non-governmental organizations to share expertise and resources to promote the growth and development of clean energy infrastructure.
6. MINT nations should prioritize education and awareness campaigns to promote the benefits of clean energy and increase public support for renewable energy adoption, and This can be achieved through public outreach programs, media campaigns, and educational initiatives to promote the importance of clean energy and its role in mitigating climate change.

By implementing these policy suggestions, MINT nations can promote developing and adopting clean energy and reduce their carbon footprint while contributing to global efforts to mitigate climate change.

Although the research provides valuable insights and contributions to the field, it is crucial to acknowledge its limitations. Below are a few potential limitations of the study: The study's objective is to establish the connections between the variables. However, due to the inherent nature of the study, establishing clear causation and directionality may pose challenges. Due to using cross-sectional data or observational analysis in the research, establishing definitive causal correlations may prove challenging. The scope of this research is limited to MINT nations, potentially restricting the generalizability of the findings to other regions or countries. The MINT nations' distinctive attributes, policies, and economic conditions may not have relevance to other regions. Therefore, it is imperative to exercise caution when extrapolating the study's conclusions beyond its specific context. This research aims to analyze multiple interconnected components within a complex system. Due to limited resources, time, and data availability, the research may be required to streamline certain aspects or exclude pertinent factors or interactions. This approximation may not fully capture the intricate interactions under consideration. By acknowledging these limitations, researchers can pinpoint areas that warrant further investigation and exercise caution when interpreting and applying the study's findings. Future studies should address the constraints above to enhance our understanding of the connections between green investment, technological innovation, financial openness, renewable energy utilization, remittances, and urbanization in MINT nations.

## Ethics approval and consent to participate

Not applicable.

## Consent for publication

“Not applicable”.

## Availability of data and materials

The datasets used and/or analyzed during the current study are available from the corresponding author on reasonable request.

## Funding

This study received financial support from the 10.13039/100007631Institute for Advanced Research (IAR), 10.13039/100019458United International University (UIU), Ref: IAR-2023-pub-025.

## CRediT authorship contribution statement

**Md Qamruzzaman:** Conceptualization, Data curation, Formal analysis, Investigation, Methodology, Writing – original draft, Writing – review & editing. **Salma Karim:** Conceptualization, Data curation, Funding acquisition, Methodology, Writing – review & editing.

## Declaration of competing interest

I, at this moment, declaring that no support from any organization for the submitted work; no financial relationships with any organizations that might have an interest in the submitted work; no other relationships or activities that could appear to have influenced the submitted work.

## List of abbreviations

GI: green investment
TI: technological innovation
FO: Financial Openness
REC: Renewable Energy Consumption
ARDL: Autoregressive Distributed Lagged
